# Comparison of the exposure of the distal tibial articular surface for posterior pilon fractures via a medial or lateral malleolar osteotomy: a preliminary cadaveric study

**DOI:** 10.3389/fsurg.2025.1702938

**Published:** 2025-12-08

**Authors:** Fan Yang, Lin Ye, Cheng Chen, Moran Huang, Yihao Sun, Zhijun Shen, Jian Hu, Lei Wang

**Affiliations:** 1 Shanghai Sixth People's Hospital Affiliated to Shanghai Jiao Tong University School of Medicine, Shanghai, China; 2 Shanghai Eighth People's Hospital, Shanghai, China

**Keywords:** distal tibial articular surface, medial malleolar osteotomy, transfibular osteotomy, comparison study, surgical exposure

## Abstract

**Objective:**

This study aimed to preliminarily investigate the exposure area of the distal tibial articular surface via a medial malleolar osteotomy or transfibular osteotomy and provide a quantitative reference for selecting a surgical approach.

**Methods:**

Five pairs of adult fresh-frozen lower extremities were included in the study, and the left and right limbs in each pair were divided randomly into either the medial approach group or the posterolateral approach group. According to the computed tomography scan, a 3D-printed guiding plate was applied to assist in the osteotomy on each specimen. The exposure area of the distal tibial articular surface was calculated after the osteotomy.

**Results:**

The average ratio of the exposed distal tibial joint dome area to the total distal tibial joint dome area for the medial and posterolateral approaches was 45.94% ± 12.79% and 47.12% ± 12.84%, respectively (S1). Moreover, intra-operative electrocautery marking (S2) yielded 62.72% ± 18.67% and 53.26% ± 10.51%, respectively. When the articular surface was subdivided into the anterior, middle, and posterior thirds, the posterior third demonstrated the greatest exposure (medial 61.3% ± 2.2%; lateral 65.2% ± 1.8%). The inter-observer intraclass correlation coefficient for S1 and S2 exceeded 0.89 (95% CI 0.81–0.97). After maximal soft tissue distraction, the exposed area further increased by 11.5 ± 2.1% (medial) and 9.1 ± 1.6% (lateral), with no significant between-group difference (*P* = 0.08, paired *t*-test).

**Conclusion:**

The medial and lateral approaches can significantly expose the distal articular surface of the posterior tibia to a large extent after medial and lateral malleolus osteotomies. A suitable individualized surgical approach conducive to adequate exposure for the operation should be considered according to the position of the main fracture fragments on the posterior articular surface and the proportion of their transverse distance to the posterior pilon variant fracture. These preliminary data require clinical validation. In addition, an intra-operative soft tissue stretch enlarges the visual field by approximately 10% regardless of the approach.

## Introduction

Ankle trauma accounts for nearly 10% of all fractures, and pilon variants that involve the weight-bearing articular surface of the distal tibia are among the most challenging subsets (1). Recent epidemiological work demonstrates that posterior pilon fractures—defined as intra-articular distal tibial injuries whose coronal fracture line reaches the posterior colliculus of the medial malleolus—now represent 7%–44% of all ankle fractures, and their incidence is rising (2, 3). The combination of vertical compressive and torsional forces that produces these injuries frequently results in comminution, marginal impaction, and intercalary osteochondral fragments (4, 5). Post-operative articular step-off as small as 2 mm has been identified as an independent predictor of poor outcomes and post-traumatic osteoarthritis, even when the fragment involves only 5%–10% of the articular surface (6–9). Computed tomography (CT)–based mapping further shows that 64% of posterior malleolar fragments are located posterolaterally, and 58% of cases contain intercalary fragments that cannot be reduced adequately through standard anterior or posterolateral windows (10, 11). Consequently, complete visualization of the posterior tibial plafond is critical, yet existing studies lack quantitative data comparing the exposure achieved by medial malleolar osteotomy vs. transfibular osteotomy (12, 13). This cadaveric study was designed to close this research gap by measuring the absolute and subregional articular surface visible through each approach and to determine how much additional exposure can be gained by maximal soft tissue distraction.

## Materials and methods

### Specimen preparation

This study included five pairs of adult lower limb specimens provided by the Shanghai Medical College of Fudan University. None of the specimens had any deformity, surgical history, trauma, tumor, or degeneration. Under direct vision and X-ray fluoroscopy, ankle joint activity was normal. Five pairs of specimens were cut from the middle leg and thawed to a natural state before the experiment. Ethical approval was obtained for this research. Details were disclosed to the journal upon submission.

All the experimental procedures for all specimens were completed by the same senior physician. The five adult lower limb specimens were dissected using either the medial approach, for an unreduced medial malleolar fracture, or the posterolateral transfibular approach, with each pair divided between groups A (medial approach) and B (posterolateral approach).

### Design of the osteotomy guide plate

Medial malleolus osteotomy guide plate: The medial malleolus osteotomy guide plate in group A was designed according to thin-layer CT and three-dimensional (3D) reconstruction data from each specimen. The osteotomy plate was placed on the medial corner of the articular surface of the distal tibia anteriorly and 0.5 cm from the tarsal tunnel posteriorly. The osteotomy plane was set at 45° from the tibiotalar articular surface of the distal tibia.

Lateral malleolus osteotomy guide plate: The lateral malleolus osteotomy guide plate in group B was designed according to thin-layer CT and 3D reconstruction data from each specimen. The osteotomy plane was established from the inferior aspect of the distal tibiofibular ligament to its superior aspect, with the anterior border of the fibula extending from the lower portion of the distal tibiofibular syndesmosis to the lowest bony point of the anterior tibia. In three-dimensional space, this plane formed a 30° angle with the longitudinal axis of the fibula and was parallel to the axial plane defined by the medial and lateral malleoli.

### Operative technique

**Figure 1:**
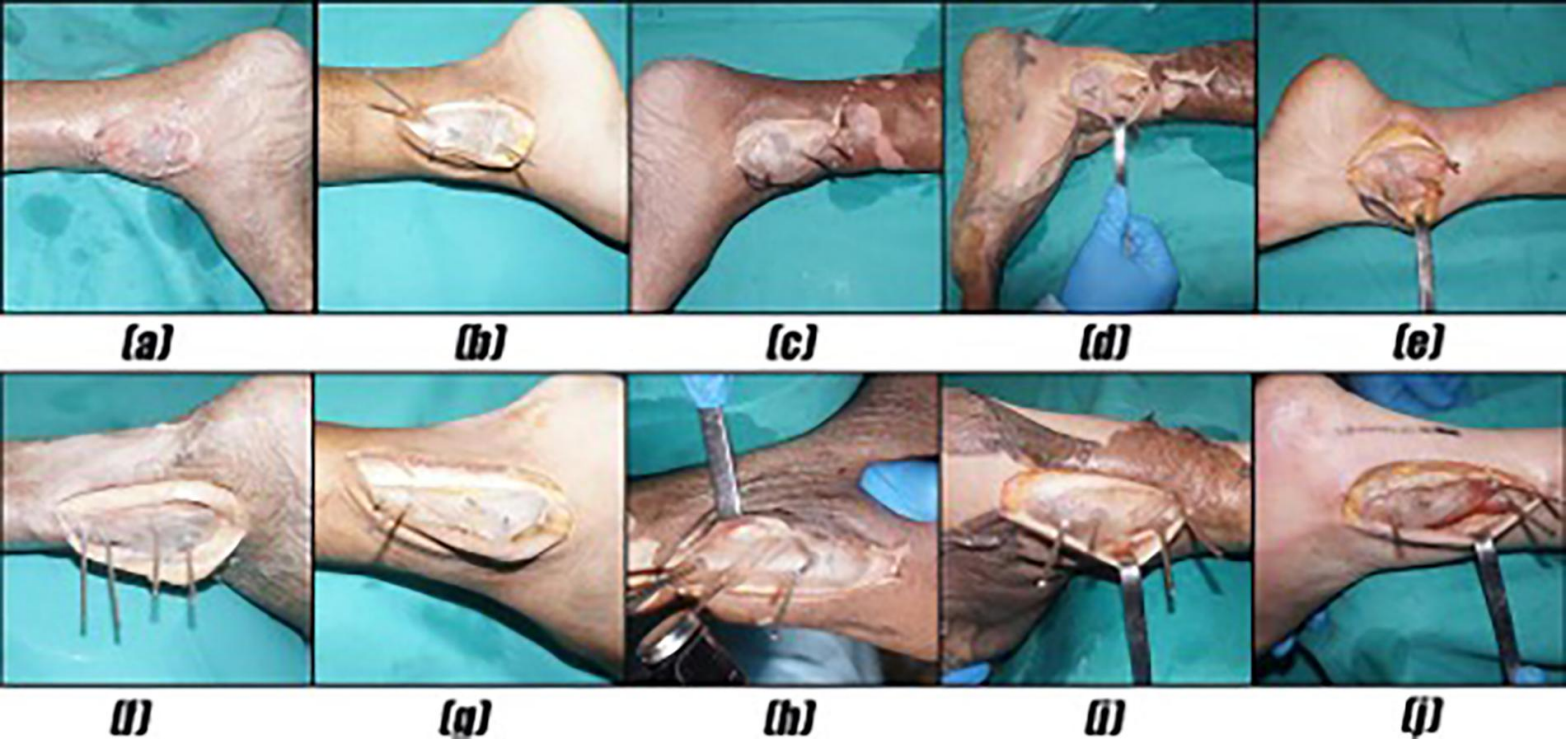
(**a**–**e**) The medial malleolus was osteotomized using an osteotomy guide plate. (**f**–**j**) The lateral malleolus was osteotomized using an osteotomy guide plate.

Medial approach: A J-shaped incision with a length of 8–10 cm was made longitudinally along the posterior edge of the medial malleolus. The medial malleolus was osteotomized using an osteotomy guide plate (Figures 1a–e). After the medial malleolar bone block was turned downward and temporarily fixed using a Kirschner wire (K-wire), an external fixation device was used laterally to maintain the valgus of the ankle joint, which simulated the exposure of the articular surface of the distal tibia through the fracture gap.

Lateral approach: An incision with a length of 8–10 cm was made longitudinally along the midline between the fibula laterally and the Achilles tendon medially, which is curved anteriorly at the distal end of the fibula. After the direct exposure of the lateral malleolus, the posterior tibial column was exposed through the interval between the peroneal tendon and the flexor hallucis longus tendon. The lateral malleolus was osteotomized using an osteotomy guide plate (Figures 1f,g). After the osteotomy gap was widened using a lamina spreader, both the proximal and distal segments were fixed to the tibia temporarily using K-wires. An external fixation device was used medially to maintain the varus of the ankle joint, which simulated the exposure of the articular surface through the fibular fracture gap.

### Measurement

**Figure 2:**
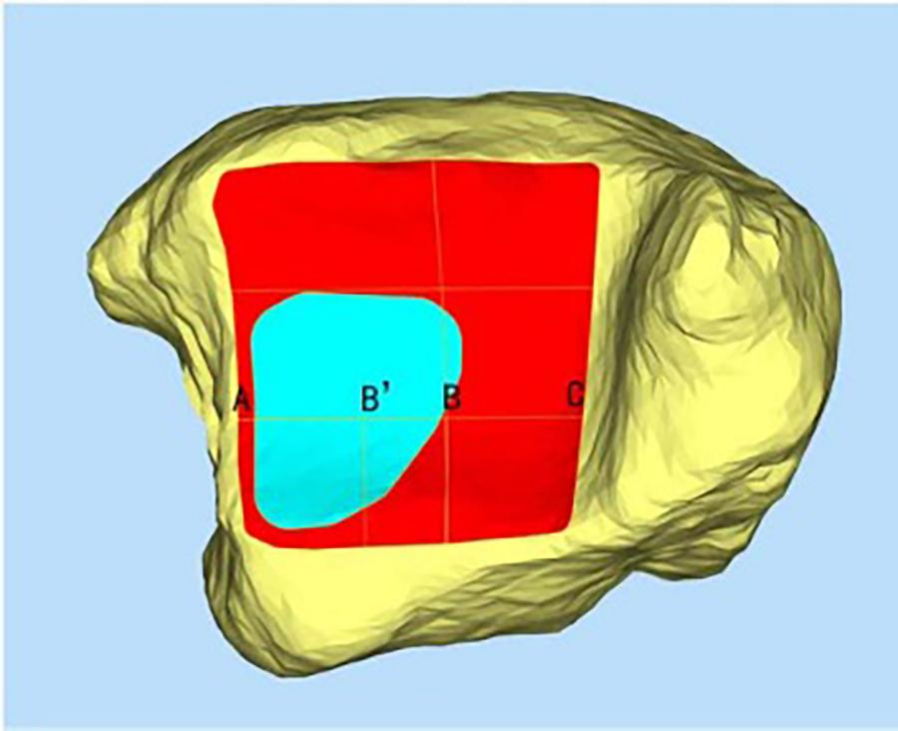
The measurement of the exposed lateral distance of the posterior third of the distal tibial articular surface (d%).

One observer measured the following four parameters three times post-osteotomy: the total distal tibial plafond area, S, which was set as the 100% reference; S1, the digitally visible articular surface through a 3D CT soft-tissue window; S2, the area directly outlined by cautery under vision; and d% (Figure 2), the lateral exposure ratio of the posterior third. This was calculated on an axial CT slice by drawing an anterior–posterior line (AC) across the posterior third, identifying the most lateral visible point (B) on AC, dropping a perpendicular line (BB′) to the visible margin, and computing (AB′/AC) × 100%, with higher d% indicating greater lateral visual access.

**Figure 3:**
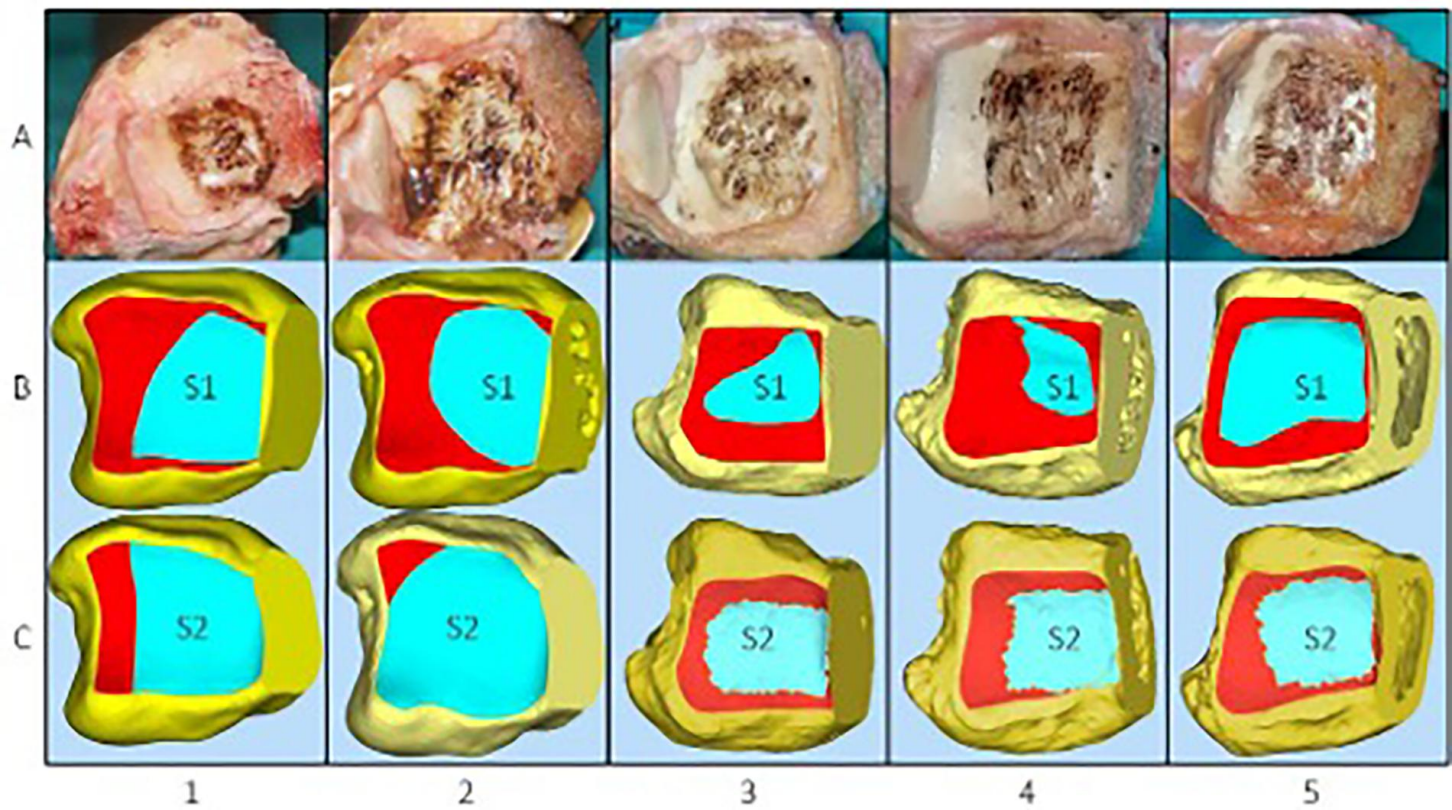
(**A**) Five specimens were dissected via a medial approach to expose the distal articular surface of the tibia following electrocautery. (**B**) Exposed area of the distal tibial articular surface (S1). (**C**) Exposed area of the distal tibial articular surface marked by electrocautery (S2).

**Figure 4:**
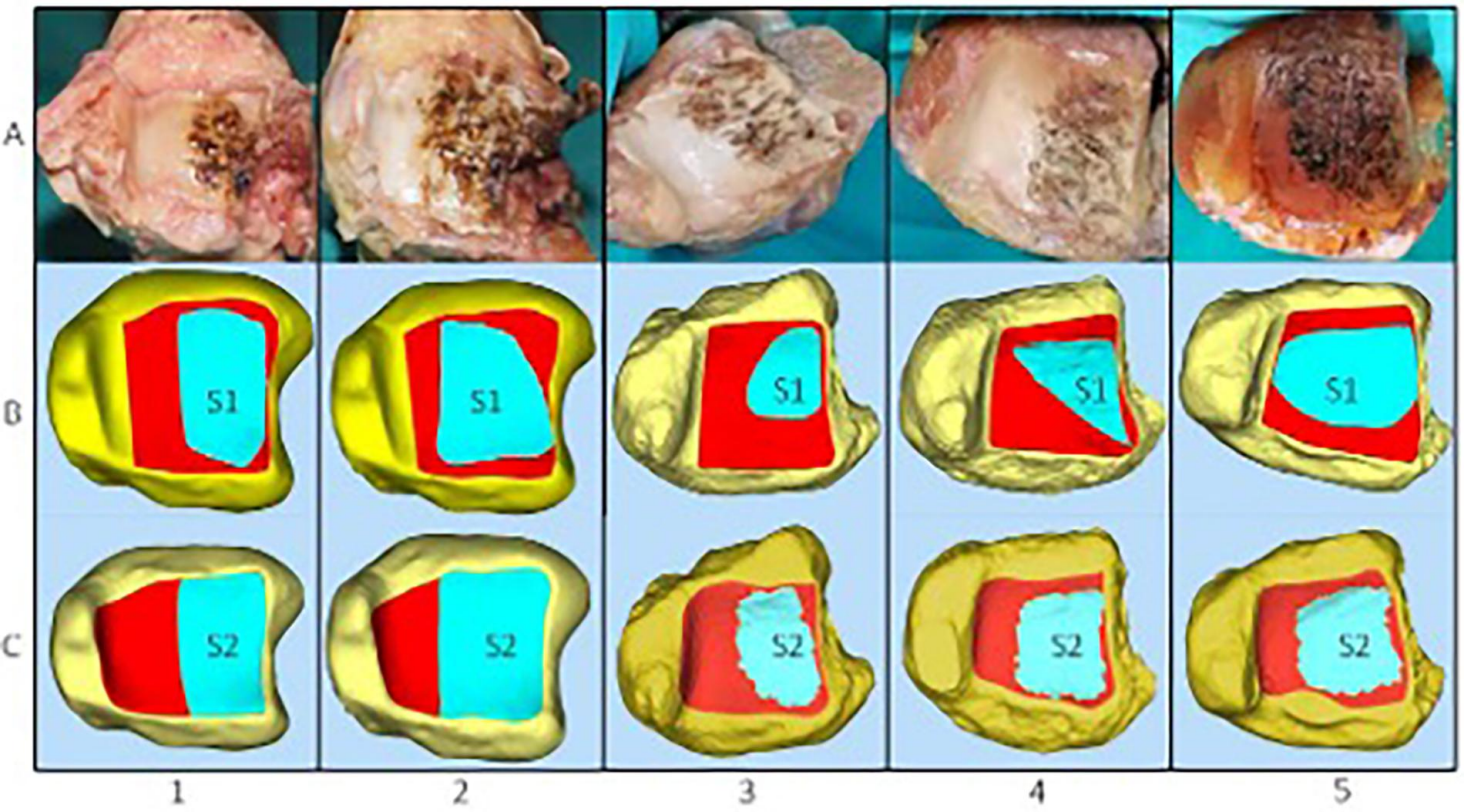
(**A**) Five specimens were dissected via a posterolateral approach to expose the distal tibial articular surface following electrocautery. (**B,C**) The exposed area of the distal tibial articular surface (S1) and the exposed area of the distal tibial articular surface marked by electrocautery (S2) were measured using Mimics software.

After the osteotomy, thin-layer CT and electrocautery marking were performed to show the range of the visible joint surface. The total area of the distal tibial articular surface (S), exposed area of the distal tibial articular surface (S1), and exposed area of the distal tibial articular surface marked by electrocautery (S2) were measured using Mimics software. Image processing was performed on raw CT data, and Mimics software was used to reconstruct bone and skin models. The bone and skin data were imported into Geomagic software and S was delineated. We then simulated the visible area of the distal tibial articular surface through a soft-tissue window and delineated S1. Finally, the data on the total area of the distal tibial articular surface and the exposed area of the distal tibial articular surface were imported into Magics to display their areas. In addition, after reconstructing the bone models using Mimics software, combined with the specimen photographs marked by electrocautery and some body surface markers, S2 was delineated in Geomagic software. The data on S2 were then imported into Magics to display the area (Figures 3, 4). Thus, the percentages of exposed area, S1% (S1% = S1/S) and S2% (S2% = S2/S), were calculated. The dividing line between the posterior and middle thirds of the articular surface of the distal tibia (AC) was measured. Point B is the intersection of the visible articular surface and the invisible articular surface on AC and was found by drawing a vertical line on AC. This point fitted the visible range of the articular surface into a rectangle, so that the area of the rectangle was equal to the area of the exposed part (Figure 2). AB′ is a part of the fitted rectangle on AC. The exposed lateral distance of the posterior third of the distal tibial articular surface was calculated by d% (d% = AB′/AC). The bone and skin data were imported into Geomagic software, and S was delineated. To quantify the line-of-sight, a 3D vector was created from the incision center to the plafond center; the angle (*α*) between this vector and the articular surface normal was calculated in 3-Matic. Each specimen was rescanned once with the osteotomy maximally distracted to assess any additional exposure (ΔS1, Δd%).

After osteotomy and provisional fixation, the surgeon used monopolar cautery, with the ankle neutral and no tourniquet, to draw three passes, i.e., circumferential rim, radial 5–7 mm spokes, and final rim, demarcating the farthest tibial plafond visible without extra retraction; the marked “true visual field” (S2) was photographed perpendicular to the joint with a 50 mm lens, registered in the 3D model via skin fiducials, and twice-segmented in Geomagic and averaged, whereas S1 is the theoretical bone exposure defined only by CT under virtual soft-tissue windows, making S1 anatomy-driven and S2 surgeon- and soft-tissue-limited.

## Statistical analysis

The statistical analyses were performed using SPSS version 22.0. An independent sample *t*-test was used to compare the percentage of the exposed area of the distal tibial articular surface according to thin-layer CT (S1%) and the percentage of the exposed area of the distal tibial articular surface marked by electrocautery (S2%) in the two groups. Statistical significance was set at *P* < 0.05. Paired *t*-tests were employed to compare the left and right legs. Inter-observer reliability was assessed using a two-way mixed intraclass correlation coefficient (ICC) (2,1). Statistical significance was set at *P* < 0.05.

## Results

Five pairs of adult lower limb specimens (10 ankles) were included. No specimen exhibited deformity, prior surgery, or degenerative change, and ankle motion was largely normal in all cases.

### Overall exposure

Using the medial approach, the mean exposed area calculated from the 3D-reconstructed model (S1) was 45.94 ± 12.79% of the entire distal tibial articular surface; the corresponding intra-operative electrocautery marking (S2) yielded 62.72 ± 18.67%. With the lateral approach, S1 was 47.12 ± 12.84% and S2 was 53.26 ± 10.51%. Paired *t*-tests showed no significant difference between approaches for S1 (*P* = 0.42), whereas S2 was significantly larger with the medial approach (mean difference 9.46%, 95% CI 2.1–16.8%, paired *t*-test *P* = 0.03) (Table 1).

**Table 1:** The exposed area of the articular surface and the exposed lateral distance of the distal tibial articular surface in the posterior third.

	Medial	Lateral
The exposed area (S1%)	45.94 ± 12.79	47.12 ± 12.84
The exposed area (S2%)	62.72 ± 18.67	53.26 ± 10.51
Paired *t*-test *p*	0.42	0.03
d%	46.42 ± 12.57	42.98 ± 7.48

S1, the average value of the exposed area of the distal tibial articular surface measured by the 3D reconstruction model; S2, the average value of the exposed area of the distal tibial articular surface marked by electrocautery; d%, the lateral proportion of the exposed part in the posterior third of the distal tibial articular surface.

### Subregional exposure

When the articular surface was divided into anterior, middle, and posterior thirds, the posterior third consistently demonstrated the greatest exposure (Table 2).

**Table 2:** Subregional exposure of the distal tibial articular surface (%, mean ± SD).

Approach	Anterior third	Middle third	Posterior third
Medial	16.2 ± 1.4	41.2 ± 1.7	61.3 ± 2.2
Lateral	13.9 ± 0.8	44.6 ± 0.9	65.2 ± 1.8

### Posterior third lateral distance (d%)

The mean proportions of the transverse distance of the exposed part within the posterior third of the articular surface were 46.42 ± 12.57% (medial) and 42.98 ± 7.48% (lateral); the paired difference was not significant (*P* = 0.08).

### Effect of maximal soft tissue distraction

After maximal distraction of the osteotomy gap, the exposed area increased by 11.5 ± 2.1% in the medial group and 9.1 ± 1.6% in the lateral group. The corresponding Δd values were 10.4 ± 1.8% and 8.0 ± 1.2%, respectively. Paired *t*-tests revealed no significant between-group difference for either ΔS1 (*P* = 0.08) or Δd (*P* = 0.11) (Table 3).

**Table 3:** Effect of maximal distraction on the exposed area (%, mean ± SD).

Approach	ΔS1 (absolute % increase)	Δd (relative % increase)
Medial	11.5 ± 2.1	10.4 ± 1.8
Lateral	9.1 ± 1.6	8.0 ± 1.2

### Measurement reliability

The two-way mixed ICC was ≥0.89 (95% CI 0.81–0.97) for S1 and ≥0.89 (95% CI 0.77–0.95) for S2, indicating excellent inter-observer reproducibility.

### Geometric parameters of the osteotomy

The mean areas of the osteotomy plane were 182 ± 11 mm^2^ (medial) and 205 ± 9 mm^2^ (lateral). The dihedral angles between the osteotomy plane and the articular surface averaged 45.0 ± 1.2° (medial) and 30.0 ± 1.0° (lateral).

### Line-of-sight angle

The average angles (*α*) between the line of sight from the incision and the articular surface normal were 28 ± 4° (medial) and 26 ± 3° (lateral), with no significant difference (*P* = 0.36).

## Discussion

Posterior pilon fractures are widely regarded as among the most technically demanding intra-articular injuries of the lower limbs, not only because of the complex osseous anatomy but also because of the restricted surgical field imposed by the dense surrounding soft tissue envelopes (1, 2). The vertical–torsional force vectors that produce these fractures frequently generate comminution, intercalary fragments, and marginal impaction that must be visualized directly if anatomical reduction is to be achieved (3, 4). Yet, until now, objective metrics describing exactly how much of the distal tibial articular surface can truly be seen through either a medial malleolar osteotomy or a transfibular osteotomy have been lacking. The current quantitative cadaveric investigation provides precisely these metrics, offering surgeons a blueprint for pre-operative decision-making.

Across all 10 specimens, the posterior third of the tibial plafond consistently emerged as the most accessible zone. Mean exposure of its surface area reached 61.3 ± 2.2% when the medial approach was employed and 65.2 ± 1.8% via the lateral approach. By contrast, the anterior third never exceeded 16%, regardless of the approach. These findings agree with recent large-scale CT reviews demonstrating that approximately two-thirds of posterior malleolar fragments are posterolateral, and that anterior extension is rare (5, 6). Consequently, both osteotomies excel at delivering the posterior facet into view, whereas supplementary anterior windows, percutaneous clamps, or arthroscopic assistance remain indispensable when fracture lines propagate anteriorly to the mid-coronal plane (7–9).

A more subtle but clinically crucial observation emerged when 3D-reconstructed data were compared with intra-operative electrocautery outlines. While the digitally calculated exposed area (S1) was statistically similar between groups, the electrocautery-based assessment (S2) revealed a 9.5% larger direct visual field through the medial malleolar fracture gap (*P* = 0.03, paired *t*-test). This discrepancy underlines the disproportionate soft tissue curtain encountered laterally; specifically, the peroneal tendons, the extensor retinaculum, and the anterior talofibular ligament create a layered barrier that narrows the cone of vision (10, 11). The 9.5% larger direct visual field (S2) through the medial osteotomy, despite equivalent bony windows (S1), is best explained by the asymmetrical soft tissue anatomy of the ankle. Medially, the flexor retinaculum and the thin capsule can be retracted *en bloc* with minimal muscle obstruction; the only significant structure is the posterior tibial tendon, which can be gently mobilized toward the plantar. Laterally, the peroneal tendons, the thick inferior extensor retinaculum, and the anterior talofibular ligament form a layered “laminated” curtain that remains tense even after adequate release of the extensor tendons. In addition, the lateral osteotomy plane (30° to fibular axis) creates an acute dihedral angle with the plafond, forcing the surgeon to look “around the corner” through a narrower soft tissue funnel. These factors reduce the effective cone of vision without altering the absolute bony aperture, producing the S2–S1 divergence we observed. Clinically, surgeons choosing the transfibular route should budget for additional flexor hallucis longus (FHL) retraction, partial peroneal tenosynovectomy, or use of an angled speculum to reclaim the 9%–10% visual deficit.

Maximal intra-operative distraction increased the visible articular surface by 9%–12% (mean 10.3%) in both cohorts, without conferring a selective advantage to either approach. The gain was non-linear, plateauing after roughly 5 mm of additional gap opening, a finding that accords with biomechanical reports showing that capsuloligamentous compliance is exhausted well before the osseous limits of the ankle mortise are reached (12, 13). Thus, while gentle distraction is always recommended, surgeons should not expect unlimited expansion; the geometry of the osteotomy itself remains the dominant determinant of the visual field.

The present study also establishes the reproducibility of the measurement protocol. Inter-observer intraclass correlation coefficients exceeded 0.89 for both S1 and S2, demonstrating that thin-slice CT, 3D reconstruction, and electrocautery marking can be performed reliably across different operators and at different time points (14). These data provide the methodological foundation for future clinical trials comparing functional outcomes against quantitative exposure metrics. Moreover, the newly reported osteotomy plane area (medial 182 ± 11 mm^2^ vs. lateral 205 ± 9 mm^2^) and dihedral angle (medial 45.0 ± 1.2° vs. lateral 30.0 ± 1.0°) can now be integrated into patient-specific cutting guides, potentially reducing angular error to less than 2° during surgery (15, 16).

Collectively, the findings support a pragmatic, fragment-oriented decision algorithm. When the posterior pilon fragment is confined to the posterolateral quadrant and does not extend medially beyond 50% of the posterior width, the transfibular approach is adequate and avoids unnecessary medial dissection. Conversely, when the fracture line or intercalary fragments encroach upon the medial malleolar region, the medial osteotomy provides a significantly larger direct visual field and facilitates plate or lag-screw placement along the medial column. Irrespective of the chosen route, pre-operative 3D planning should incorporate an expected line-of-sight angle of 26°–28° to ensure that all the critical fragments fall within the visual cone, thereby reducing the risk of residual step-off and late arthrosis (17).

Several limitations must be acknowledged. Cadaveric specimens lack the edema, callus, and capsular scarring found in live patients, so the absolute visual field may differ, and functional outcomes such as reduction accuracy, implant prominence, and long-term arthrosis were not assessed. Although the observed 9.5% difference in S2 reached statistical significance (*P* = 0.03) in the current sample of five matched pairs, future studies aiming to replicate this effect with 90% power should consider increasing the sample size to approximately 14–16 specimens per group, based on *post-hoc* sample size calculations. These quantitative exposure data should therefore be regarded as preliminary, and larger cadaveric series together with prospective clinical trials that correlate exposure metrics with reduction accuracy, implant prominence, and patient-centered outcomes are required before definitive surgical algorithms can be established.

## Conclusion

In the absence of soft-tissue edema or post-traumatic scarring, both medial and lateral malleolar osteotomies deliver substantial visual access to the posterior tibial plafond, with the posterior third being the most exposed region. Based on the present cadaveric data, we propose a threshold of 50% of the posterior articular width: when the fracture extends medial to this line, medial malleolar osteotomy provides a significantly larger visual field (9.5%, *P* = 0.03). Conversely, fractures confined to the lateral half can be adequately managed via the transfibular approach. If an associated fibular or medial malleolar fracture is present, the existing fracture gap may be used instead of a formal osteotomy, although this scenario awaits clinical validation.

## Data Availability

The raw data supporting the conclusions of this article will be made available by the authors, without undue reservation.
